# Revisiting Neutrophil Gelatinase-Associated Lipocalin (NGAL) in Cancer: Saint or Sinner?

**DOI:** 10.3390/cancers10090336

**Published:** 2018-09-18

**Authors:** Brigitte Bauvois, Santos A. Susin

**Affiliations:** 1 INSERM UMRS 1138, Cell Death and Drug Resistance in Lymphoproliferative Disorders Team, Centre de Recherche des Cordeliers, 75006 Paris, France; santos.susin@crc.jussieu.fr; 2 Sorbonne Universités Paris Cité, F-75006 Paris, France; 3 Université Paris Descartes, F-75005 Paris, France

**Keywords:** cancer, drug resistance, invasion, migration, matrix metalloproteinase-9, neutrophil gelatinase-associated lipocalin, signaling, survival

## Abstract

Human neutrophil gelatinase-associated lipocalin (NGAL) is a glycoprotein present in a wide variety of tissues and cell types. NGAL exists as a 25 kDa monomer, a 46 kDa homodimer (the most abundant form in healthy subjects) and a 130 kDa disulfide-linked heterodimer bound to latent matrix metalloproteinase-9. Dysregulated expression of NGAL in human malignancies suggests its value as a clinical marker. A growing body of evidence is highlighting NGAL’s paradoxical (i.e., both beneficial and detrimental) effects on cellular processes associated with tumor development (proliferation, survival, migration, invasion, and multidrug resistance). At least two distinct cell surface receptors are identified for NGAL. This review (i) summarizes our current knowledge of NGAL’s expression profiles in solid tumors and leukemias, and (ii) critically evaluates the beneficial and detrimental activities of NGAL having been documented in a diverse range of cancer-derived cell lines. A better understanding of the causal relationships between NGAL dysregulation and tumor development will require a fine analysis of the molecular aspects and biological role(s) of NGAL both in primary tumors and at different stages of disease. Having an accurate picture of NGAL’s contribution to tumor progression is a prerequisite for attempting to modulate this protein as a putative therapeutic target.

## 1. Introduction

Human neutrophil gelatinase-associated lipocalin (NGAL) is a 25 kDa glycosylated protein from the lipocalin family [1]. The lipocalins’ common secondary and tertiary structure corresponds to a single, eight-stranded antiparallel β-barrel around a central pocket that is capable of binding low-molecular-weight ligands [1]. NGAL was initially characterized as an antibacterial immune factor via the pocket’s ability to capture siderophores (such as bacterial enterochelin and mammalian endogenous catechols) that bind iron with high affinity, causing iron depletion and thus the inhibition of bacterial cell growth [1,2]. A 30 kDa isoform of NGAL has been described, and probably results from differential glycosylation [3]. NGAL also exists as a 46 kDa disulfide-linked homodimer (the most abundant form in healthy subjects) and a 130 kDa heterodimer bound to the inactive zymogen form of the matrix metalloproteinase-9 (proMMP-9) [1,4]. Indeed, NGAL was first purified from human neutrophils because of its ability to bind proMMP-9 into a disulfide-linked complex [5]; the Cys-87 in NGAL forms a disulfide bond with an as yet unidentified cysteine residue in MMP-9’s hemopexin domain [1]. Excellent reviews have described the genomic organization of the NGAL gene and the protein’s three-dimensional structure, as well as its expression profiles in adult and fetal tissues, and biological fluids [1,4,6].

There is now evidence to suggest that NGAL may be a marker of disease status in chronic and acute pathological conditions in general and in inflammatory, metabolic, neurologic and cancer diseases in particular [2,4,7,8,9,10]. For example, urine and blood levels of NGAL monomer increase following acute kidney injury (nephron epithelia damage) [7,11,12].

The initial functional studies investigated the role of lipocalin-2 (Lcn-2, the murine homolog of human NGAL) in a mouse model [1,13,14]. However, Lcn-2 exhibits little homology with human NGAL (62%), and notably does not contain the unpaired cysteine that can form the NGAL homodimer and the NGAL-proMMP-9 heterodimer in humans [1]. These facts are crucial when analyzing the specific roles attributed to NGAL in humans, which might be distinct from that of Lcn-2 in mice [1,2,4,13,14]. For example, human NGAL is not involved in myeloid cell apoptosis or acute response in contrast to what was previously reported for Lcn-2 in mice [15]. A growing number of studies have explored the NGAL’s possible roles in various models of cancer, and suggest that the protein has both beneficial and detrimental functions [1,2,4,14]. Although ongoing studies are investigating the value of the NGAL-proMMP-9 complex as a marker of disease status in cancer, there are still no detailed data on its full functional significance in this disease [1,16].

Here, we briefly review the current literature on NGAL’s expression profiles (both free and complexed to proMMP-9) in solid tumors and leukemias. With regard to its dysregulation in cancers, NGAL could represent a promising molecular target for therapy in cancer. However, the road to pharmacological targeting of NGAL is not straightforward. The challenge now facing researchers and clinicians is to definitively understand how cells utilize NGAL in the context of tumor progression. The intention of this article is: (i) to evaluate the positive or negative effects of free NGAL observed in various models of cancer by focusing on several aspects that have not been considered before (investigative techniques used to study NGAL’s role, NGAL isoforms, NGAL receptors); (ii) to consider the remaining challenges and; (iii) to discuss the prospects for determining NGAL’s functional value in cancer.

## 2. Methods

Data and references from relevant articles were identified by searches of the electronic database PubMed, using the search terms ‘‘neutrophil gelatinase-associated lipocalin”, “NGAL”, ‘‘lipocalin”, ‘‘Lcn-2’’, “cancer”, “solid tumor”, ‘‘neoplasma”, “hematological disease”, “leukemia”, ‘‘pronostic marker’’, ‘‘diagnostic marker’’, and ‘‘review’’. Only articles published in English between 1993 and 2018 were included. The last search was run in August 2018. Studies on animal models and with murine Lcn-2 were excluded. One author (B.B.) first performed the literature search and the selection of the eligible papers (based on title and abstract), and then reviewed the full texts of all potentially eligible studies for final inclusion. The eligibility criteria included: (i) studies that evaluated NGAL as prognostic or diagnostic marker in cancer; (ii) studies that evaluated the in vitro functions of NGAL in human cancer cell models; and (iii) internationally renowned and referenced reviews. With regard to the relevant publications, both authors collected data about study design, type of cancer, methodologies to study NGAL’s roles. No disagreement appeared between authors.

## 3. NGAL as a Biomarker in Cancer

NGAL is regularly expressed in a large variety of cell types including adipocytes, hepatocytes, pneumocytes, splenocytes, mesangial and microglial cells, renal epithelial cells, and vascular smooth muscle cells [1,4]. Concerning the immune system, during hematopoiesis, immature (CD34^+^) bone marrow progenitor cells express NGAL [17]. During the maturation of granulocyte precursors in the bone marrow, NGAL is synthesized almost exclusively by myelocytes and metamyelocytes [18]. Expression of NGAL and its 130 kDa complex is also observed in activated monocytes and neutrophils [5,17], and the dimer is the major molecular form of free NGAL secreted by neutrophils [19]. To date, NGAL protein expression has never been reported in resting B and T lymphocytes. Circulating low levels of the protein (mainly as the dimer) are detected in the urine and blood of healthy subjects [19,20]. The main sources of circulating NGAL are thought to be neutrophils and renal epithelial cells [7,19].

Below, we summarize current knowledge of NGAL dysregulation in cancer (Table 1).

### 3.1. Solid Tumors

A number of recent reviews have reported NGAL dysregulation in cancer [1,4,13,14,25]. Quantitative measurements of the NGAL protein and mRNA levels performed in blood, urine and tissues, show that NGAL is overexpressed in non-microbe-associated cancers (including breast, brain, ovarian, endometrial, pancreatic, colorectal, bladder, liver, and lung cancers) [1,4,13,14,25,28,33,36]. In striking contrast, one study shows that NGAL is downregulated in primary malignant and metastatic tissues of oral cancer compared to normal tissues [46].

The abnormally elevated levels of NGAL in most cancers appear significantly correlated with disease severity and poor survival [1,4,7,8,9,14,21,25]. For example, studies on breast cancer suggest the usefulness of serum NGAL in monitoring disease progression [22] and the association of serum NGAL with reduced survival [23]. NGAL appears to be a diagnostic biomarker of advanced or recurrent ovarian cancer [27] and pancreatic cancer [36]. Although serum NGAL levels increase in patients with colorectal cancer [30,31], NGAL does not seem to be suitable as a diagnostic biomarker [30], but can have a prognostic utility in metastatic patients [31,32]. NGAL levels in gastric tumor tissues are associated with worse survival [40]. NGAL is not useful for diagnosing renal cell carcinoma [37], but it may be helpful to select a proper therapy in cases of metastatic disease without the need for pretreatment biopsy [38]. Interestingly, Roli et al. recently reevaluated the potential value of NGAL as a prognostic and diagnostic marker in cancer [47]. Their meta-analysis shows that high NGAL levels in biological fluids, such as serum and urine, could be useful to predict disease-free survival for patients with colorectal and breast cancer, but its prognostic and diagnostic accuracy remains uncertain for other human tumors, including pancreatic, thyroid, liver, lung, esophageal, oral, and kidney tumors [47]. Note that in the cancers evaluated, the inclusion of single studies enrolling a limited number of patients influence the results by overestimating the effect size [47].

Few studies investigated the value of the 130 kDa NGAL complex as a marker of disease status in several solid tumors [22,24,34,37,39,41]. The expression of the NGAL complex often correlates with the aggressive behavior of gastric, anaplastic thyroid, breast, kidney, and oral cancer cells [22,37,39,40,41]. In endometrial cancer, the NGAL complex may be useful in the assessment of tumor stage before surgical treatment [29].

### 3.2. Leukemias

Acute and chronic lymphoid/myeloid leukemias are clonal disorders that result from the neoplastic transformation of hematopoietic progenitor cells. These diseases are characterized by the survival and expansion of clonal progenitors, cell dissemination from the bone marrow into the blood and peripheral tissues, and often resistance to chemotherapy [48,49,50,51].

More precisely, acute lymphoblastic leukemia (ALL) is a heterogeneous disease that includes B and T-ALL cancers [48]. Chronic lymphocytic leukemia (CLL) is characterized by the accumulation of monoclonal B lymphocytes (CD19^+^, CD5^+^) in the peripheral blood, bone marrow, and secondary lymphoid organs [49]. Acute myeloid leukemia (AML) is a highly heterogeneous disease characterized by the clonal expansion and accumulation of hematopoietic stem cells arrested at various stages of development [50]. Chronic myeloid leukemia (CML) is a clonal myeloproliferative disorder that originates from a pluripotent stem cell expressing the Ph chromosome (t(9;22) chromosomal translocation) with a constitutively active BCR-ABL fusion gene, leading to the production of the p210 BCR-ABL protein [51].

The NGAL complex is found in blood tumor cells from patients with ALL, AML and CLL types of leukemia [16,42,44,45]. Overexpression of free NGAL is observed in blood cells from patients with all types of leukemia [16,25,42,44,45]. Paradoxically, the levels of bone marrow NGAL transcript are found to be lower in AML patients than in healthy individuals, and these levels recover to normal values following complete remission and then decline again at relapse [43]. In patients with AML, higher bone marrow NGAL mRNA expression is observed in individuals with a good prognosis than in individuals with a poor prognosis, and independently of the French-American-British (FAB) classification [43]. Moreover, patients with higher expression levels of NGAL mRNA in the bone marrow in combination with the wild type *FLT3*-ITD sequence have better prognoses [43]. The prognostic or predictive value of NGAL (dimer and/or monomer, free and complexed) in the serum of patients with AML, ALL, or CLL remains to be determined. With regard to CML, inhibition of the constitutive tyrosine kinase activity of p210 BCR-ABL [51] with imatinib alleviates the hyperproliferation-induced symptoms [52]. Furthermore, serum levels of NGAL are significantly higher in CML patients than in healthy individuals [44,45,53,54]. If CML patients achieve complete molecular remission after imatinib therapy, NGAL serum levels fall and are significantly lower than during the full-blown disease [44,53]. These findings indicate that NGAL is a useful marker of CML’s response to treatment, and strongly suggest the existence of a functional link between NGAL and BCR-ABL.

## 4. Detrimental and Beneficial Effects of NGAL in Cancer

In normal tissues, NGAL serves to provide protection against bacterial infection and modulate oxidative stress [1]. Few studies have investigated NGAL’s physiological function(s) in the hematopoietic system by treating cells with recombinant human NGAL (rhNGAL) [15,55,56,57]. NGAL does not appear to influence the balance between survival and death of bone marrow stem and progenitor CD34^+^ cells, mature granulocytes, and T and B lymphocytes [15,55], whereas it blocks the maturation of lineage-committed myeloid cells into mature erythrocytes and monocytes [55]. In contrast, a study performed by Lu et al. [56] suggests that NGAL induces the death of bone marrow CD34^+^ cells through the production of reactive oxygen species. Moreover, it seems that NGAL may favor the differentiation of bone marrow and mesenchymal stem cells into osteoblasts and fibroblasts, respectively [56]. Another study demonstrated that NGAL increases the number of T-regulatory cells (Tregs) in the peripheral blood mononuclear cell population by up-regulating the human leukocyte antigen G (HLA-G) complex, which is a mediator of tolerance [57]. This observation may have important implications for cancer, since a number of preclinical and clinical studies have linked the presence of Tregs to an increased risk of carcinogenesis and cancer development [58].

Several investigative techniques have been used to study NGAL’s role in tumor models: (i) treatment of cells with rhNGAL or neutralizing NGAL antibodies (NGAL Abs), and (ii) stable overexpression or knockdown (using siRNA) of NGAL expression with sense or antisense NGAL cDNA.

Current knowledge of NGAL’s divergent functions in cancer is summarized in Table 2.

In different models of human cancer (lung, thyroid, gastric, and breast cancer), NGAL facilitates the survival and proliferation of malignant cells [24,59,60,61,62,63]. In lung carcinoma, NGAL might protect against oxidative stress by activating the nuclear factor E2-related factor 2/heme oxygenase-1 (Nrf2/HO-1) pathway [60] and inducing the expression of heme oxygenase-1 and superoxide dismutase 1,2 [64]. Paradoxically, NGAL inhibits the proliferation and invasion of liver carcinoma cells, and this inhibition is associated with the blockade of the JNK and PI3/Akt signaling pathways [65]. In a model of advanced pancreatic cancer, NGAL reduced invasion (by suppressing FAK activation) and inhibited angiogenesis (by blocking VEGF production) [66]. In contrast, NGAL increased the motility and invasion of colon carcinoma cells by modifying the subcellular localization of E-cadherin and Rac1 (one of the Rho small GTPases) through an iron-dependent mechanism [67]. These data are consistent with reports in which NGAL favors the migration and invasion of endometrial cancer and cholangiocarcinoma cells [63,68].

Several research groups have already analyzed NGAL’s role in multidrug resistance [43,46,63,69,70,71,72,73]. While NGAL does not interfere with doxorubicin resistance in breast and colorectal cancer cells [73], it might favor the intracellular accumulation of other chemotherapeutic drugs in breast cancer [28], renal cancer [71], glioblastoma [70], oral squamous cancer [46] and leukemic AML [43] cell lines. In contrast, elevated NGAL levels might contribute to drug resistance in endometrial [63] and non-small cell lung [69] cancer cells.

Finally, whether NGAL bound to proMMP-9 retains a function has not yet been established. It has been suggested that NGAL, by forming the NGAL complex, could protect proMMP-9 from proteolytic degradation [20] and/or support its allosteric activation [74]. By binding to cell surface receptors, NGAL and proMMP-9 can initiate signal transducing events that control tumor cell processes [16]. It is therefore legitimate to suggest that the NGAL complex could interfere with the binding of NGAL and/or proMMP-9 to their respective receptors, thus modulating signaling events induced by free NGAL and/or proMMP-9.

**Figure 1:**
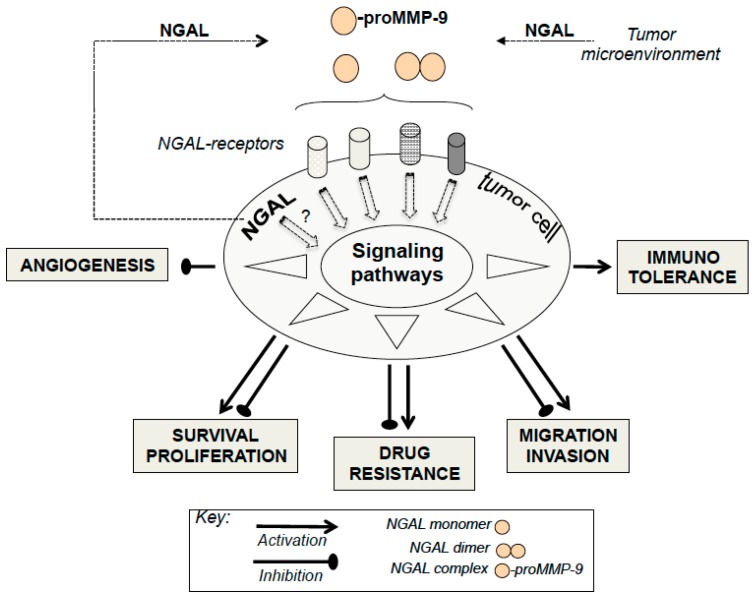
Schematic Diagram Illustrating the Putative Roles of neutrophil gelatinase-associated lipocalin (NGAL) in Modulating Major Cellular Processes. The synthesis and release of tumor NGAL (monomer, dimer, or complex) increase in response to various stimuli (inflammatory cytokines, hypoxia etc.). Extracellular NGAL binds to specific cell surface receptors (megalin, SLC22A7 isoforms) on tumor cells, and thus may activate or inactivate signaling pathways. In turn, this modulates proliferation, survival, migration, invasion, angiogenesis, immunotolerance, and multidrug resistance—all events involved in tumor biology. A given cell type may be involved in the NGAL-mediated actions reported here to a variable extent. Intracellular NGAL might be directly involved in the modulation of cell responses. The NGAL complex’s possible effects on tumor cells remain to be identified.

Thus, NGAL appears to exhibit negative or positive effects on tumor progression, depending on the type of cancer in question, as shown in Figure 1. Consequently, these multifaceted roles of NGAL observed in the pathophysiology of cancer may compromise the potential therapeutic application of any NGAL inhibition or stimulation proposed by several authors [1,13,75,76,77,78]. Currently, apart from anti-NGAL antibodies, no specific inhibitors (or inducers) of NGAL are commercially available. In our opinion, before developing NGAL-targeted therapy, a major challenge requires an accurate evaluation of NGAL effects in certain cancers.

## 5. Conclusions and Future Directions

In molecular terms, there is increasing evidence of crosstalk between NGAL and cancer. There is a growing body of evidence suggesting that NGAL overexpression in tumors results from stimuli (including hypoxia and inflammatory cytokines) present in the tumor microenvironment [77]. The NF-κB signaling pathway, activated in most cancers including leukemias, regulates the transcription of NGAL [1] and the MAPK pathway may cooperate with NF-κB to up-regulate the expression of NGAL [1]. Moreover, epigenetic modifications might be important in initiating NGAL expression in the tumor cells. This may explain the increased levels of NGAL in most cancers. It remains to identify the specific molecular forms of NGAL (in serums and in cells) associated with a specific type of cancer (solid or liquid). In functional terms, as seen in Figure 1, NGAL appears to exhibit either beneficial or detrimental effects through the modulation of proliferation, survival, migration, invasion, angiogenesis, and drug resistance—all cellular events considered to be hallmarks of cancer [79]. How, then, can NGAL’s opposing effects in cells be explained? One may consider that the one or more functional roles of NGAL expressed by tumor cells are intrinsically linked to a specific type of cancer. However, several parameters have to be taken into account when assessing NGAL’s roles in malignant cells. Firstly, all of the above-mentioned functional studies used cancer-derived cell lines that might not reflect the progressively malignant stages of a given cancer. Secondly, it is possible that our understanding of NGAL’s pro-/anti-tumor activities has been conditioned by the different technical approaches used in the studies. Indeed, NGAL overexpression and silencing probably target intracellular NGAL localization, whereas rhNGAL and NGAL antibodies respectively mimic and target extracellular NGAL. As seen in leukocytes, inside-out and outside-in signaling mechanisms often overlap [80]. Alternatively, NGAL’s actions inside and outside cells may be exerted through distinct mechanisms. For example, the study by Tong et al. [59] indicated that inhibiting intracellular NGAL expression with siRNA in A549 lung cancer cells increases cell death, whereas cell treatment with rhNGAL, even at high doses, had no effect on cell viability. Finally, in the functional studies using rhNGAL, the molecular form of rhNGAL (monomer or dimer) and the NGAL receptor involved in the cellular events have not been characterized; this might also explain NGAL’s divergent effects.

Two distinct cell surface receptors were initially identified for NGAL: low-density lipoprotein receptor-related protein-2 (LRP-2, also known as megalin) [81] and the solute carrier family 22 member 17 (also known as SLC22A17 and NGAL-receptor 2) [82]. LRP-2 internalizes a variety of unrelated ligands, including nutrients, hormones and their carrier proteins, signaling molecules, and extracellular proteins [83]. Both LRP-2 and NGAL-R2 bind both free and iron-bound NGAL, and promote the latter’s endocytosis [1,81,82]. Two splice variants of SLC22A17 (designated as NGAL-R1 and NGAL-R3) have also been identified, and appear to be involved in NGAL-mediated transport inside cells [84]. It remains to be determined whether all these receptors are able to bind all the different forms of NGAL (monomer, dimer and complex). Accordingly, the paradoxical effect of rhNGAL on the survival of bone marrow CD34^+^ cells might possibly be related to molecular differences between the distinct sources of rhNGAL [15,56]. Moreover, the existence of distinct NGAL receptors suggests that the signaling pathways through which cellular responses are induced might be distinct or similar. These key signaling pathways might be involved singly or concurrently in tumor progression. Importantly, the NGAL receptors have not been characterized in most cancers—including leukemias. For each type of cancer studied, characterization of NGAL’s molecular forms, receptors, and related signaling mechanisms will be essential for gaining a better understanding of the role of tumor NGAL in cancer progression. Furthermore, greater knowledge of the intracellular signaling pathways induced by NGAL might provide a molecular basis for targeted therapeutic approaches.

Novel therapies are needed to overcome resistance to chemotherapeutic drugs, and the identification of novel, eligible cancer targets is always of general interest. Given the abnormally high levels of NGAL secreted in disease settings, it has been suggested that NGAL is a therapeutic target. Few studies have investigated the role of NGAL in cancer drug resistance, as shown in Table 2. Unexpectedly, NGAL contributes to both drug resistance and sensitivity, depending on the chemotherapeutic drug and the type of cancer in question. These findings therefore provide the rationale for further investigations of NGAL putative utility as a drug target.

In conclusion, dysregulation of NGAL expression is observed in tumor cells. NGAL now appears to be a pleiotropic cytokine, but an essential question for clinicians and scientists is whether NGAL participates to the disease process as a ‘‘saint’’ or a ‘‘sinner’’. The challenge is now to solve all the above-mentioned questions and thus establish an integrated model of NGAL’s actions in cancer.

## Figures and Tables

**Table 1:** Potential value of neutrophil gelatinase-associated lipocalin (NGAL) as a biomarker in solid tumors and leukemias.

Cancer Type	NGAL	NGAL Complex	Ref.
Breast	serum/tissue	serum/urine	[1,4,10,13,14,21,22,23,24,25,26]
Brain	tissue		[1,4,13]
Ovary	serum/urine/tissue		[1,13,25,27]
Endometrium	serum/tissue	serum	[4,13,28,29]
Colorectal	plasma/serum/tissue		[1,4,13,30,31,32]
Bladder	serum/urine/tissue	serum/urine	[25,33]
Prostate		urine	[34]
Liver	tissue		[4,25,35]
Lung	tissue		[1,2,35]
Pancreas	plasma/serum/tissue		[1,4,25,36]
Kidney	serum/urine/tissue	serum/urine	[1,25,35,37,38]
Esophagus	tissue		[4,25,35,39]
Gastric	serum/tissue		[25,35,40]
Thyroid	tissue		[1,41]
ALL	cell	cell	[16,25]
CLL	cell	cell	[16,25,42]
AML	cell	cell	[16,25,43]
CML	plasma/serum/cell		[1,16,42,44,45]

**Table 2:** Beneficial and detrimental effects of neutrophil gelatinase-associated lipocalin (NGAL) in cancer.

Cancer Type	Investigative Technique	Impact of NGAL on Cell Processes	Ref.
Lung (A549)	siRNA anti-NGAL	Survival ↑ Oxidative stress ↓	[59]
Lung (A549, PC9)	siRNA	Proliferation ↑ Oxidative stress ↓	[60]
Lung and liver (A549, HepG2)	siRNA NGAL overexpression	Oxidative stress ↓	[64]
Anaplastic thyroid (FRO)	rhNGAL (*), siRNA anti-NGAL	Survival ↑	[61]
Gastric (MGC-803, SGC-7901)	siRNA	Survival & Proliferation ↑	[62]
Liver (SK-Hep-1)	rhNGAL (R&D) NGAL overexpression	Proliferation & Migration ↓	[65]
Pancreatic (Paca)	siRNA NGAL overexpression	Invasion & Angiogenesis ↓	[66]
Colon (KM12C, HCT116, DLD1)	siRNA NGAL overexpression	Cell-cell adhesion ↓ Migration ↑	[67]
Colon (KM12SM)	NGAL overexpression	Invasion ↓	[76]
Cholangiocarcinoma (RMCCA-1)	siRNA	Migration & Invasion ↑	[68]
Endometrial (HHUA, RL95-2)	siRNA NGAL overexpression	Survival & Migration ↑ Cisplatin resistance ↑	[63]
Squamous cell carcinoma (SAS)	siRNA	Survival & Migration ↓ Cisplatin Resistance ↓	[46]
Non-small-cell lung (H441, H3255)	siRNA NGAL overexpression	Erlotinib Resistance ↑	[69]
Glioblastoma (U87MG, U373MG, T98G)	NGAL overexpression	Carmustine Resistance ↓ Cell Death ↑	[70]
Renal cancer (Caki1, A498, ACHN)	rhNGAL (Pfizer, Sigma) NGAL overexpression	Sunitinib Resistance ↓	[71]
Breast (MDA-MB-23)	rhNGAL (R&D, Sino Biological Inc.) siRNA	Rhodamine-123 Resistance ↓	[72]
Breast (MCF-7)	NGAL overexpression	Proliferation & Angiogenesis ↑ No effect on Doxorubicin Resistance	[24,73]
Colorectal (HT-29)	NGAL overexpression	No effect on Doxorubicin Resistance	[73]
AML (THP1, OCI-AML3)	NGAL overexpression	Cytarabine Resistance ↓ Cell death ↑	[43]

rhNGAL: recombinant human NGAL; ROS, reactive oxygen species; * not commercially available rhNGAL; Nrf2: nuclear factor E2-related factor-2. Cisplatin and cytarabine inhibit DNA replication; carmustine (1,3-bis(2-chloroethyl)-1-nitrosourea) and doxorubicin prevent DNA replication and transcription; erlotinib is an EGFR tyrosine kinase inhibitor; sunitinib is a multi-targeted receptor tyrosine kinase inhibitor. ↑ stimulation; ↓ inhibition.

## Abbreviations

The following abbreviations are used in this manuscript:

AML	acute myeloid leukemia
ALL	acute lymphoblastic leukemia
CLL	chronic lymphocytic leukemia
CML	chronic myeloid leukemia
FAB	French-American-British
HLA-G	human leukocyte antigen G
Lcn-2	ipocalin-2
LRP-2	low-density lipoprotein receptor-related protein-2
Neutrophil gelatinase-associated lipocalin	NGAL
NGAL Ab	NGAL antibody
NGAL-R	NGAL-receptor
Nrf2/HO-1	nuclear factor E2-related factor 2/heme oxygenase-1
proMMP-9	pro-matrix metalloproteinase-9
rhNGAL	recombinant human NGAL
ROS	reactive oxygen species
Nrf2	nuclear factor E2-related factor-2
SLC22A17	solute carrier family 22 member 17
Treg	T-regulatory cell
